# Synthesis and Selective Cytotoxic Activities on Rhabdomyosarcoma and Noncancerous Cells of Some Heterocyclic Chalcones

**DOI:** 10.3390/molecules21030329

**Published:** 2016-03-09

**Authors:** Tuong-Ha Do, Dai-Minh Nguyen, Van-Dat Truong, Thi-Hong-Tuoi Do, Minh-Tri Le, Thanh-Quan Pham, Khac-Minh Thai, Thanh-Dao Tran

**Affiliations:** 1 Faculty of Applied Sciences, Ton Duc Thang University, 19 Nguyen Huu Tho St., Tan Phong Ward, Dist. 7, Ho Chi Minh City 700000, Vietnam; dotuongha@tdt.edu.vn; 2 Faculty of Pharmacy, University of Medicine and Pharmacy, Ho Chi Minh City, 41 Dinh Tien Hoang St., Dist. 1, Ho Chi Minh City 700000, Vietnam; nguyendaiminh.medchem@gmail.com (D.-M.N.); dattv.vn@gmail.com (V.-D.T.); hongtuoid99@gmail.com (T.-H.-T.D.); leminhtri1099@gmail.com (M.-T.L.); 3 Faculty of Chemical Engineering, Ho Chi Minh City University of Technology, Vietnam National University, Ho Chi Minh City, 268 Ly Thuong Kiet St, Dist. 10, Ho Chi Minh City 700000, Vietnam; ptquan@hcmut.edu.vn

**Keywords:** heterocyclic chalcones, cytotoxicity, rhabdomyosarcoma, LLC-PK1 cell, MTT

## Abstract

Chemically diverse heterocyclic chalcones were prepared and evaluated for cytotoxicity, aiming to push forward potency and selectivity. They were tested against rhabdomyosarcoma (RMS) and noncancerous cell line (LLC-PK1). The influence of heteroaryl patterns on rings A and B was studied. Heterocycle functionalities on both rings, such as phenothiazine, thiophene, furan and pyridine were evaluated. Notably, the introduction of three methoxy groups at positions 3, 4, 5 on ring B appears to be critical for cytotoxicity. The best compound, with potent and selective cytotoxicity (IC_50_ = 12.51 μM in comparison with the value 10.84 μM of paclitaxel), contains a phenothiazine moiety on ring A and a thiophene heterocycle on ring B. Most of the potential compounds only show weak cytoxicity on the noncancerous cell line LLC-PK1.

## 1. Introduction

Statistics indicate that cancer is the second most frequent cause of death in the U.S. only after cardiovascular disease and the leading cause of death in the UK [1,2]. Some of the dominant drawbacks of present anticancer drug therapy involve their lack of significant greater toxicity towards cancer cells in comparison with normal tissue and the rise of multi-drug resistance. Tumor-selective cytotoxic agents whose structures are sufficiently different from anticancer medication currently on the market are therefore highly sought.

Soft tissue sarcomas constitute a heterogeneous group of neoplasms which accounts for approximately 7% of all cancer cases in patients under the age of 20 [3,4,5]. According to pediatric oncologists, these tumors are arbitrarily divided into two groups: rhabdomyosarcoma (RMS), the most common soft tissue sarcomas in children less than 10 years of age, and non-RMS soft tissue sarcomas (NRSTS), predominating in the older age groups [3].

Chemotherapy using well-known anticancer medicines including vinscristine, dactinomycin and cyclophosphamide is mostly indicated for all patients with RMS [3,6]. Other agents such as melphalan, methotrexate, doxorubicin, cisplatin, ifosfamide, toptecan and irenotican are active against RMS, but in combination chemotherapy. To date, none of them have been shown to improve outcome [3].

Chalcones represent an important group of the flavonoid family, which includes a large number of naturally occurring and synthetic molecules. The chemical structure of chalcones consists of two aromatic rings joined by a three carbon, α,β-unsaturated carbonyl system (1,3-diphenylprop-2-en-1-one) [7]. They have been documented with diverse biological activities including antibacterial [8,9,10,11,12,13,14], anti-inflammatory [15,16,17], antioxidant [18,19,20,21], anti-tumor effects [22,23,24,25,26,27]. Recent studies have demonstrated that chalcones are absorbed in the daily diet and appear to be promising as potential chemopreventive and chemotherapeutic compounds [28]. Although the availability of heterocyclic chalcones from natural sources is limited, they have been reported to possess a wide range of bioactivities, especially cytotoxic activity [29]. Compared to the current cancer drugs, chalcones have the advantages of being inexpensive, available and less toxic. In addition, the ease of synthesis also makes them an attractive drug scaffold. Thus, the identification of new heterocyclic chalcones with anticancer activity against cancerous cells including RMS is of great interest.

**Figure 1:**
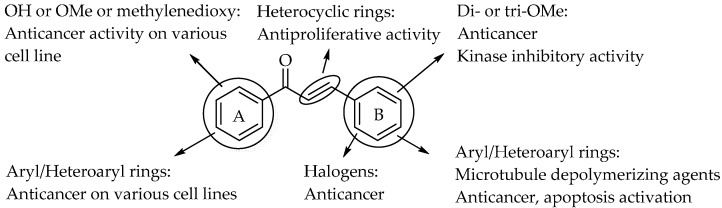
Structure- anticancer activity relationship of chalcone compounds [30].

Very recently, the structure-activity relationship of cytotoxic chalcone compounds were established based on the systematic review of literature from 2007 to 2014 [30]. The general results are summarized in Figure 1. Supported by these conclusions, we herein carry out the structural modification of both aryl rings, including the replacement of aryl rings with heteroaryl moieties and introduction of methoxy groups on rings A and B for enhancement of anticancer properties.

All synthesized chalcones were evaluated for their cytotoxicity on RMS cells by a MTT (3-(4,5-dimethylthiazolyl-2)-2,5-diphenyltetrazolium bromide) cell proliferation assay. Additionally, a similar assay was conducted on a noncancerous cell line (renal tubule cells, LLC-PK1) to identify the most selective anticancer agents.

## 2. Results and Discussion

### 2.1. Chemistry

**Scheme 1:**
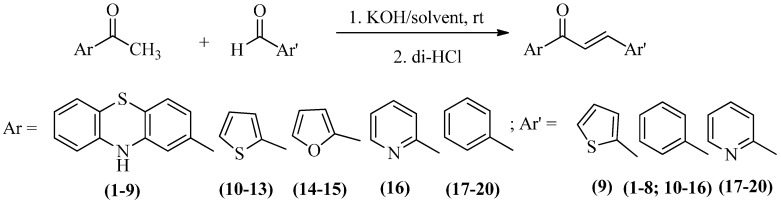
General key step for the synthesis of heterocyclic chalcones.

The synthesis of all the target compounds was accomplished by classical Claisen-Schmidt condensation between an acetophenone derivative and a corresponding aryl aldehyde in methanolic/KOH at room temperature, as depicted in Scheme 1 [31,32,33]. This aldol condensation via a one-step reaction produced five new substituted chalcones **1**, **2**, **3**, **7**, **8** and **9** with phenothiazine moieties and fifteen known derivatives **4**–**6**, **10**–**20**, as listed in Table 1 and Table 2. To our knowledge, the chemical data of the new compounds and some others have not been described in the literature until now, so their spectral data is presented herein for the first time.

### 2.2. Evaluation of Cytotoxic Activities

The MTT assay for determination of cell viability was adapted from previously described procedures [35]. The cytotoxicity assay results are shown in Table 3 and the cytotoxic profile of the potent synthesized heterocyclic chalcones on noncancerous cells (LLC-PK1) is displayed in Table 4.

Overall, the studied heterocyclic chalcone compounds demonstrate low to strong cytotoxic properties on RMS cells, in which compounds **7**, **8**, **9** and **16** exhibit promising potentials with IC_50_ values below 20 µM. These results are in accordance with reported intitial structure-activity relationship of anticancer chalcones, *i.e.*, heteroaryl moieties either on ring A or ring B of chalcones provide cytotoxicity on cancer cells [30]. More interestingly, compounds **7**, **8**, **9** and **16** show an almost complete absence of toxicity on the noncancerous LLC-PK1 cell line.

For chalcones with a phenothiazine moiety, substitution on the B-ring by halogen gave variable results: a single Cl at position 2 was found to increase cytotoxicity greatly (**7**), whereas a single Cl at position 4 or Br atom at position 3 (**3** and **4**) brought limited effect as compared to their phenyl counterpart (**5**). Moreover, phenothiazinyl chalcones with other functional groups displayed insignificant cytotoxicity. Notably, the presence of a thiophene ring led to the most potent chalcone in our series (**9**). From this observation, it is apparent that an electronegative center at the position 2 of ring B enhance cytotoxicities greatly. Besides, the presence of two methoxy groups at positions 2 and 4 on ring B followed the same trend as that of electronegative groups.

In terms of chalcones with ring B replaced with pyridine moieties (typically chalcones **19** and **20** in comparison with **17** and **18**), a methoxy group at position 4′ on ring A also enhances the cytotoxic properties. This has emphasized the role of alkoxy group at position 4′ of 2′-hydroxychalcones in exhibiting the cytotoxic profile.

The activities of 3,4,5-trimethoxy-containing chalcone analogues (**13**, **14** and **16**) are consistent with what has been hypothesized. Additionally, the order of heterocycles’ contribution to cytotoxicity is as followed: pyridine > thiophene > furan.

More interestingly, chalcone **14** shows its cytotoxicity ascending on proliferation inhibition 15.1%, 26.5% and 42.9% when decreasing concentrations of 100 μM, 50 μM and 25 μM, respectively. This led us to speculate that the action mode of these heterocyclic chalcones was similar to that of colchicine and combrestatin A-4, mostly by the similarity in their structure. The planar structure of heterocycles pyridine, furan and thiophene may be responsible for the cytotoxic properties.

## 3. Experimental Section

### 3.1. General Information

All reagents and chemicals were obtained from commercial sources, and used without further purification. Melting points were determined on open capillary tubes and are uncorrected (using a Gallenkamp apparatus). UV spectra were measured on a Hitachi U-2010 instrument (Hitachi High-Technologies, Tokyo, Japan). ^1^H- and ^13^C-NMR spectra were recorded on a Bruker Avance II instrument at 500 MHz and 125 MHz, respectively (Bruker Corporation, Billerica MA, USA). Chemical shifts are reported in parts per million (ppm) downfield relative to tetramethylsilane as an internal standard. Peak splitting patterns are abbreviated as m (multiplet), s (singlet), brs (broad singlet), d (doublet), bd (broad doublet), t (triplet) and dd (doublet of doublets). MS spectra were acquired on an Agilent LC-MS 1200 Series instrument (Agilent Technologies, Santa Clara, CA USA) with MS detector of micrOTOF-QII Bruker. Thin layer chromatography (TLC) used Merck silicagel F-254 plates (thickness = 0.25 mm). Column chromatopraphy used silica gel 60, 320–400 mesh.

### 3.2. General Procedure for the Synthesis of Chalcones ***1**–**20***

A solution of heteroaryl methyl ketone (5 mM) and aryl/heteroaryl aldehyde (5 mM) in 15 mL of solvent (DMSO for chalcones **1**–**9**; methanol for the other compounds **10**–**20**) was cooled to 5–10 °C in an ice bath. The cooled solution was treated with a small portion of pulverized KOH (10 mM). The reaction mixture was magnetically stirred for 60 min and then left overnight or longer and monitored by thin layer chromatography using hexane–acetone (5:1) as developing solvent. The resulting dark solution was diluted with ice water and carefully acidified using dilute hydrochloric acid. The chalcones, which separated as a yellow solid, were collected by filtration after washing with water and further purified by crystallization from appropriate solvents. The crude product was further purified by column chromatography if necessary. Coupling constants (*J*) from the proton nuclear magnetic resonance (^1^H-NMR) spectra clearly indicated that all synthesized chalcone analogues were geometrically pure and were exclusively *trans* (*E*) isomers with *J*C_2_=C_3_ calculated in the range of 15–16 Hz.

### 3.3. Product Characterization

*(E)-3-(4-(N,N-Dimethylamino)phenyl)-1-(10H-phenothiazin-2-yl)prop-2-en-1-one* (**1**). Yield 45%. Mp: 232–233 °C. EIMS *m*/*z*: 371.1286 [M − H]^−^. UV (λ_max_ nm, MeOH): 204, 248, 292, 425. IR (KBr) cm^−1^: 3314, 1650, 1600. ^1^H-NMR (DMSO) δ ppm: 8.75 (s, ^1^H, NH), 7.67–7.64 (d, 1H, *J*_3-2_ = 15.5 Hz, H3), 7.65–7.63 (d, 2H, *J*_2″-3″_ = *J*_6″-5″_ = 9Hz, H2″, H6″), 7.55 (d, *J*_3′-4′_ = 7.5 Hz, 1H, H3′), 7.47 (d, *J*_2-3_ = 15.5 Hz, 1H, H2), 7.30 (s, 1H, H1′), 7.05 (d, *J*_4′-3′_ = 7.5 Hz, 1H, H4′), 7.00 (t, *J*_8′-7′_ = *J*_8′-9′_ = 7.5 Hz), 6.92 (d, *J*_6′-7′_ = 7.5 Hz, 1H, H6′), 6.76 (t, *J*_7′-6′_ = 7.5 Hz, 1H, H7′), 6.74 (d, *J*_3″-2″_ = 9 Hz, *J*_5″-6″_ = 9Hz, 2H, H3″, H5″), 6.67 (d, *J*_8′-9′_ = 7.5 Hz, 1H, H9’), 3.00 (s, 6H, NMe_2_). ^13^C-NMR (DMSO) δ ppm: 187.4 (C1=O), 151.9 (C4″), 144.9 (C3), 142.0 (C13’), 141.2 (C11″), 137.7 (C2’), 130.6 (C2″, C6″), 127.9 (C8′), 126.2 (C6′), 126.0 (C4′), 122.5 (C1″), 122.0 (C7′), 122.0 (C2), 121.9 (C3’), 115.8 (C1′), 115.3 (C12′), 114.5 (C14’), 112.9 (C9′), 111.7 (C3″, C5″), 39.63 (NMe_2_).

*(E)-3-(4-Benzyloxyphenyl)-1-(10H-phenothiazin-2-yl)prop-2-en-1-one* (**2**). Yield 55%. Mp: 215–216 °C. EIMS *m*/*z*: 436.2321 [M − H]^−^. UV (λ_max_ nm, MeOH): 204, 247, 303. IR (KBr) cm^−1^: 3366, 1653, 1571, 1257. ^1^H-NMR (DMSO) δ ppm: 8.76 (s, 1H, NH), 7.81 (d, *J*_2″-3″_ = 8.5 Hz, *J*_5″-6″_ = 8.5 Hz, 2H, H2″, H6″), 7.69 (d, *J*_3-2_ = 15.5 Hz, 1H, H3), 7.64 (d, *J*_2-3_ = 15.5 Hz, 1H, H2), 7.59 (dd, *J*_3′-4′_ = 8 Hz, *J*_3′-1′_ = 1.5 Hz, 1H, H3′), 7.47 (d, *J*_2‴-3‴_ = 7.5 Hz, *J*_6‴-5‴_ = 7.5 Hz, 2H, H2‴, H6‴), 7.41 (dd, *J*_3‴-2‴_ = 7.5 Hz, *J*_3‴-4‴_ = *J*_3‴-5‴_ = 7.5 Hz, *J*_5‴-6‴_ = 7.5 Hz, 2H, H3‴, H5‴), 7.34 (t, *J*_4__‴-3‴_ = *J*_4__‴-5‴_ = 7.5 Hz, 1H, H4‴), 7.30 (d, *J*_1′-3′_ = 1.5 Hz, 1H, H1′), 7.1 (d, *J*_3”-2”_ = 8.5 Hz, *J*_5”-6”_ = 7.5 Hz, 2H, H3”, H5”), 7.07 (d, *J*_3′-4′_ = 8 Hz, 1H, H4′), 7.01 (m, 1H, H8′), 6.92 (d, *J*_6′-7′_ = 7.5 Hz, 1H, H6′), 6.77 (m, 1H, H7′), 6.66 (d, *J*_9′-8′_ = 7.5 Hz, 1H, H9′), 5.18 (s, 2H, CH_2_O). ^13^C-NMR (DMSO) δ ppm 187.8 (C1=O), 160.4 (C4″), 143.7 (C3), 142.1 (C13′), 141.1 (C11′), 137.11 (C2′), 136.7 (C1‴), 130.6 (C2″, C6″), 128.4 (C3‴, C5‴), 127.9 (C8′), 127.9 (C2‴, C6‴), 127.7 (C4‴), 127.5 (C1″), 126.2 (C6′), 126.0 (C4′), 123.2 (C7′), 122.3 (C2), 122.0 (C1′), 119.4 (C3′), 115.2 (C12′, C14′), 114.5 (C9′), 112.9 (C3″, C5″), 69.4 (CH_2_O).

*(E)-3-(3-Bromophenyl)-1-(10H-phenothiazin-2-yl)prop-2-en-1-one* (**3**). Yield 43%. Mp: 230–232 °C. EIMS *m*/*z*: 405.9936 [M − H]^−^. UV (λ_max_ nm, MeOH): 245, 307, 450. IR (KBr) cm^−1^: 3349, 1651, 1587, 582. ^1^H-NMR (DMSO) δ ppm 8.78 (s, 1H, NH), 8.17 (s, 1H, H2″), 7.87–7.85 (d, *J*_3-2_ = 15.5 Hz, 1H, H3), 7.83 (d, *J*_6″-5″_ = 7.5 Hz, 1H, H6″), 7.67–7.64 (d, 1H, *J*_2-3_ = 15.5 Hz, H2), 7.66 (d, *J*_4″-5″_ = 8Hz, 1H, H4″), 7.63 (d, *J*_3′-4′_ = 8 Hz, 1H, H3′), 7.41 (t, 1H, H5″), 7.30 (s, 1H, H1′), 7.08 (d, *J*_4′-3′_ = 8 Hz, 1H, H4′), 7.01 (m, 1H, H8′), 6.92 (d, *J*_6′-7′_ = 7.5 Hz, 1H, H6′), 6.77 (m, 1H, H7′), 6.66 (d, *J*_9′-8′_ = 8 Hz, 1H, H9′). ^13^C-NMR (DMSO) δ ppm: 187.8 (C1=O), 142.1 (C3), 141.9 (C13′), 141.0 (C11″), 137.2 (C1″), 136.6 (C2′), 133.0 (C2″), 130.9 (C4″), 130.7 (C5″), 128.1 (C8′), 128.0 (C6″), 126.2 (C6′), 126.1 (C4′), 123.9 (C3″), 123.4 (C7′), 122.7 (C3′), 122.3 (C1′), 122.1 (C2), 115.1 (C12′), 114.6 (C14′), 112.9 (C9′).

*(E)-3-(2-Chlorophenyl)-1-(10H-phenothiazin-2-yl)prop-2-en-1-one* (**7**). Yield 57%. Mp: 202–203 °C. EIMS *m*/*z*: 386.0331 [M + Na]^+^. UV (λ_max_ nm, MeOH): 204, 248, 309, 449. IR (KBr) cm^−1^: 3354, 1654, 1590, 754. ^1^H-NMR (DMSO) δ ppm: 8.79 (s, 1H, NH), 8.16 (d, *J*_3″-4″_ = 7.5 Hz, 1H, H3″), 8.00 (d, *J*_3-2_ = 15.5 Hz, 1H, H3), 7.84 (d, *J*_2-3_ = 15.5 Hz, 1H, H2), 7.64 (d, *J*_3′-4′_ = 8 Hz, 1H, H3′), 7.57 (d, *J*_6″-5″_ = 7.5 Hz, 1H, H6″), 7.46 (m, 2H, H4″, H5″), 7.30 (s, 1H, H1′), 7.09 (d, *J*_4′-3′_ = 8 Hz, 1H, H4′), 7.01 (m, 1H, H8′), 6.92 (d, *J*_6′-7′_ = 7.5 Hz, 1H, H6′), 6.66 (m, 1H, H7′), 6.66 (d, *J*_9′-8′_ = 8 Hz, 1H, H9′). ^13^C-NMR (DMSO) δ ppm: 187.7 (C1=O), 142.1 (C3), 141.0 (C13′), 138.2 (C11′), 136.5 (C2′), 134.2 (C2″), 132.2 (C1″), 131.9 (C3″), 130 (C4″), 128.4 (C8′), 127.9 (C6″), 127.6 (C5′), 126.2 (C5″), 126.1 (C4′), 124.5 (C7′), 124.4 (C2), 122.7 (C3′), 115.1 (C12′), 114.5 (C14′), 112.8 (C9′).

*(E)-3-(2,4-Dimethoxyphenyl)-1-(10H-phenothiazin-2-yl)prop-2-en-1-one* (**8**). Yield 49%. Mp: 178–179 °C. EIMS *m*/*z*: 388.1154 [M − H]^−^. UV (λ_max_ nm, MeOH): 204, 248, 304, 365. IR (KBr) cm^−1^: 3310, 1643, 1570, 1272, 1150. ^1^H-NMR (DMSO) δ ppm: 8.76 (s, 1H, NH), 7.95 (d, *J*_3-2_ = 15.5 Hz, 1H, H3), 7.86 (d, *J*_6″-5″_ = 8.5 Hz, 1H, H6″), 7.61 (d, *J*_2-3_ = 15.5 Hz, 1H, H2), 7.52 (dd, *J*_3′-4′_ = 8 Hz, *J*_3′-1′_ = 1.5 Hz, 1H, H3′), 7.28 (d, *J*_1′-3′_ = 1.5 Hz, 1H, H1′), 7.06 (d, *J*_4′-3′_ = 8 Hz, 1H, H4′), 7.01 (m, 1H, H8′), 6.92 (d, *J*_6′-7′_ = 7 Hz, 1H, H6′), 6.80 (m, 1H, H7′), 6.64 (m, 3H, H9′, H3″, H5″), 3.90 (s, 3H, 2″-OMe), 3.84 (s, 3H, 4′-OMe). ^13^C-NMR (DMSO) δ ppm: 187.9 (C1=O), 163.1 (C4″), 160 (C2″), 142.1 (C13′), 141.2 (C11′), 138.6 (C3), 137.3 (C2′), 130.1 (C6″), 127.9 (C8′), 126.2 (C6′), 126.1 (C4′), 123.0 (C7′), 122.1 (C2), 122.0 (C3′), 118.9 (C1″), 115.9 (C1′), 115.2 (C12′), 114.5 (C14′), 112.9 (C9′), 106.3 (C5″), 98.3 (C3″), 55.8 (2′-MeO), 55.5 (4′-CH_3_O).

*(E)-3-(Thiophen-2-yl)-1-(10H-phenothiazin-2-yl)prop-2-en-1-one* (**9**). Yield 60%. Mp: 229–230 °C. EIMS *m*/*z*: 334.0042 [M − H]^–^. UV (λ_max_ nm, CH_2_Cl_2_): 249, 340. IR (KBr) cm^−1^: 3342, 1645, 1601. ^1^H-NMR (DMSO) δ ppm: 8.78 (s, 1H, NH), 7.89 (d, *J*_3-2_ = 15 Hz, 1H, H3), 7.79 (d, *J*_4″-5″_ = 5 Hz, 1H, H5″), 7.67 (d, *J*_3″-4″_ = 3.5 Hz, 1H, H3″), 7.50 (dd, *J*_3′-4′_ = 8 Hz, *J*_3′-1′_ = 1.5 Hz, 1H, H3′), 7.40 (d, *J*_2-3_ = 15 Hz, 1H, H2), 7.27 (d, *J*_1′-3′_ = 1.5 Hz, 1H, H1′), 7.19 (dd, *J*_4″-3″_ = 3.5 Hz, *J*_4″-5″_ = 5 Hz, 1H, H4″), 7.07 (d, *J*_4′-3′_ = 8 Hz, 1H, H4′), 7.00 (m, 1H, H8′), 6.92 (dd, *J*_6′-7′_ = 8 Hz, 1H, H6′), 6.77 (m, 1H, H7′), 6.66 (dd, *J*_9′-7′_ = 1.5 Hz, *J*_9′-8′_ = 1.5 Hz, 1H, H9′). ^13^C-NMR (DMSO) δ ppm: 187.4 (C1=O), 142.1 (C13′), 141.1 (C11′), 139.7 (C2″), 136.8 (C2′), 136.6 (C3), 132.9 (C5″), 130.4 (C3″), 128.7 (C4″), 128.0 (C8′), 126.2 (C2), 126.2 (C6′), 123.4 (C4′), 122.2 (C7′), 120.0 (C1′), 115.2 (C12′), 114.6 (C14′), 112.9 (C9′).

*(E)-3-(Pyridin-2-yl)-1-(2-hydroxyphenyl)prop-2-en-1-one* (**17**). Yield 52%. Mp: 136–138 °C. EIMS *m*/*z*: 226.0861 [M − H]^+^. IR (KBr) cm^−1^: 1641, 1577. ^1^H-NMR (CDCl_3_), δ ppm: 12.72 (s, 1H, OH); 8.8 (s, 1H, H2-pyridine), 8.24–8.27 (d, 1H, *J*_3-2_ = 15 Hz, H3), 8.03-8.05 (dd, 1H, *J*_6″-4″_ = 1.5 Hz, *J*_6″-5″_ = 8 Hz, H6″), 7.83–7.86 (d, 1H, *J*_2-3_ = 15 Hz, H2), 7.74–7.77 (t, 1H, H4″), 7.47–7.51 (dd, 1H, H3′-pyridine), 7.30–7.32 (t, 1H, H4′-pyridine), 7.02–7.04 (d, 1H, H3″), 6.93–6.96 (t, 1H, H5′-pyridine). ^13^C-NMR (CDCl_3_), δ ppm: 193.1 (C1=O), 163.7 (C2″), 151.4 (C2′, C6′), 150.1 (C2′), 141 (C3), 136.7 (C4″), 134.7 (C4′), 130.4 (C3′), 129.7 (C6″), 123.8 (C2), 122.1 (C5′), 119.8 (C5″), 119 (C1″), 118.8 (C3″).

*(E)-3-(Pyridin-3-yl)-1-(2-hydroxyphenyl)prop-2-en-1-one* (**18**). Yield 65%. Mp: 120–122 °C. EIMS *m*/*z*: 226.0852 [M − H]^+^. ^1^H-NMR (CDCl_3_), δ ppm: 12.62 (s, 1H, OH); 8.9 (s, 1H, H2′-pyridine); 8.66–8.67 (d, 1H, H6′-pyridine); 7.98–7.99 (d, 1H, *J* = 7.5 Hz, H4′-pyridine); 7.89–7.92 (d, 1H, 7.5 Hz, H6″); 7.88–7.91 (d, 1H, *J*_3-2_ = 16 Hz, H3); 7.72–7.75 (d, 1H, *J*_2-3_ = 16 Hz, H2); 7.51–7.54 (t, 1H, *J* = 1.5 Hz, 8.5 Hz, H4″); 7.39–7.42 (t, 1H, *J* = 5 Hz, 8 Hz, H5-pyridine); 7.04-7.06 (d, 1H, *J* = 8.5 Hz, H3″); 6.59–6.50 (t, 1H, *J* = 7.5 Hz, H5″). ^13^C-NMR (CDCl_3_), δ ppm: 193.1 (C1=O); 163.7 (C2″); 151.4 (C4′-pyridine); 150.1 (C2′-pyridine); 141.5 (C3); 136.7 (C4″); 134.7 (C6′-pyridine); 130.4 (C1′-pyridine); 129.6 (C6″); 123.8 (C2); 122.1 (C5′-pyridine); 119.8 (C5″); 119 (C1″); 118.7 (C3″)

*(E)-3-(Pyridin-2-yl)-1-(2-hydroxy-4-methoxyphenyl)prop-2-en-1-one* (**19**). Yield 65% over two steps. Mp 130–131 °C. EIMS *m*/*z*: 256.0861 [M + H]^+^. ^1^H-NMR (CDCl_3_), δ ppm: 13,36 (s, 1H, OH), 8.68–8.69 (m, 1H, H3′), 8.17 (d, *J*_3-2_ = 15 Hz, 1H, H3), 7.94 (d, *J*_5″-6″_ = 8.5 Hz, 1H, H6″), 7.81 (d, *J*_2-3_ = 15 Hz, 1H, H2), 7.75 (dt, *J*_5-6_ = *J*_5-4_ = 7.5 Hz, *J*_5-3_ = 1.5 Hz, 1H, H5′), 7.46 (d, *J*_6-5_ = 7.5 Hz, 1H, H6′), 7.27–7.31 (m, 1H, H4′), 6.47–6.50 (m, 2H, H3″, H5″), 3.86 (s, 3H, OCH_3_). ^13^C-NMR (CDCl_3_), δ ppm: 192 (C1=O), 166.7 (C4″), 166.4 (C2″), 153 (C1′), 150.2 (C3′), 142.2 (C3), 136.9 (C5′), 131.8 (C6″), 125.6 (C2), 124.5 (C6′), 124.4 (C4′), 114.2 (C1″), 107.7 (C5″), 101 (C3″), 55.61 (OCH_3_).

*(E)-3-(Pyridin-3-yl)-1-(2-hydroxy-4-methoxyphenyl)prop-2-en-1-one* (**20**). Yield 60% over two steps. Mp 128–129 °C. EIMS *m/z*: 256.0962 [M − H]^+^. ^1^H-NMR (DMSO), δ ppm: 13.32 (s, 1H, OH), 9.03 (s, 1H, H2′-pyridine), 8.61–8.62 (m, 1H, H4′-pyridine), 8,36 (d, *J*_6″-5″_ = 8 Hz, 1H, H6″), 8.28 (d, *J*_6′-5′_ = 8 Hz, 1H, H6′-pyridine), 8.13 (d, J3-2 = 15.5 Hz, 1H, H3), 7.83 (d, *J*_2-3_ = 15,5 Hz, 1H, H2), 7.49 (dd, *J*_5′-6′_ = 8 Hz, 1H, H5′), 6.57 (dd, *J*_5″-6″_ = 8 Hz, *J*_5″-3″_ = 2,5 Hz, 1H, H5″), 6.52 (d, *J*_3″-5″_ = 2.5 Hz, 1H, H3″), 3,85 (s, 1H, OCH_3_). ^13^C-NMR (DMSO), δ ppm: 191.4 (C1=O), 166.1 (C4″), 165.7 (C2″), 151 (C4′), 150 (C2′), 140.5 (C3), 135.2 (C6′), 132.7 (C6″), 130.6 (C1′), 123.8 (C2), 123.1 (C5′), 113.7 (C1″), 107.5 (C5″), 100.9 (C2″), 55.7 (OCH_3_).

### 3.4. MTT Assay of Cytotoxic Activity

Human muscle rhabdomyosarcoma (RMS) cell line (ATCC^®^ CCL-136™) was provided by World Health Organization (WHO) for The Institute of Pasteur in Ho Chi Minh City, Vietnam according to the Poliovirus Monitoring Project protocols.

Pig Kidney Epithelial (LLC-PK1) cell line (ATCC^®^ CL-101™) was a generous gift of Prof. Jeong Hill Park, Research Institute of Pharmaceutical Science (College of Pharmacy, Seoul National University, Seoul 151-172, Korea).

RMS or LLC-PK1 cells were seeded and cultured in EMEM medium (Gibco, Waltham, MA, USA) supplemented with 10% fetal calf serum (FCS) (Gibco), 2 mM l-glutamine, 100 IU/mL penicillin and 100 µg/mL streptomycin (Sigma-Aldrich, St. Louis, MO, USA) in a humidified atmosphere of 5% CO_2_ at 37 °C.

The samples were dissolved in dimethyl sulfoxide (DMSO) at 10 mM and then diluted with culture medium to get three concentrations of 100; 50; 10 µM or 25; 12.5; 2.5 µM for cell proliferation assays. The final concentration of DMSO in culture medium was 1%; 0.5% or 0.1% (*v*/*v*), which did not affect cell viability [35]. DMSO at different concentrations or was used as a blank control. Paclitaxel (Anzatax^®^, Mayne Pharma, Australia) was used as the reference compound at the concentration of 1, 5 and 10 µM (directly dissolved in culture medium). 

The cells were cells were seeded in 96-well plates at 4.2 × 10^4^ cell/cm^2^. After 24 h of incubation at 37 °C, 5% CO_2_, 100 μL of chalcone samples at tested concentrations, reference compound, and blank control (DMSO) was added and incubated for 24 hours at 37 °C, 5% CO_2_.

Cell viability was evaluated as mitochondrial succinate dehydrogenase (SDH) activity, a marker of viable cells, using MTT test as described by Denizot and Lang [36]. Briefly, SDH activity was detected after 3 hours incubation in culture medium without FCS containing 0.05 mg/mL MTT (3-4,5-dimethyl-thiazol-2-yl-2,5 diphenyl-tetrazolium bromide, Sigma-Aldrich), which is converted into formazan dissolved in acidified isopropanol (Merck, Darmstadt, Germany) by agitation for 10 min at room temperature. The produced purple solution was spectrophotometrically measured at 570 nm on Multiskan™ FC microplate Photometer (Thermo Scientific, Waltham, MA, USA).

Each assay was done in triplicate and the standard deviation of absorbance was less than 10% of the mean. The results were expressed as Mean + SD of six cultures of one representative experiment and statistically analyzed by Mann-Whitney test with SPSS 22.0 for Windows software. Threshold of significance was set at *p* < 0.05.

The percentage of proliferation inhibition was calcutated as follows:

% Proliferation inhibition = 100 − [(A_sample_/A_blank control_) × 100] (1)

The IC_50_ values were calculated based on the linear regression between the concentrations of testing compound and the inhibition percentages of cells. The chalcone compounds with significant potential against RMS cells (IC_50_ < 20 μM) were then tested for their toxicity on LLC-PK1 cells.

## 4. Conclusions

Several lines of investigation suggest that by replacing the original phenyl ring of chalcones by heteroaryl moieties, resulting heterocyclic chalcone compounds may be effective anticancer agents. The study presented herein demonstrated that some representative heterocyclic chalcones, namely, phenothiazinyl chalcones and pyridinyl 2′-hydroxychalcones exhibit promising anticancer properties. Thus, these classes of chalcones, whose structures are far different from anticancer drugs currently on the market, exhibit potential cytotoxicity. This study will provide further assistance to the design of more potent and selective cytotoxic chalcones and will allow us to correlate the structural features with the biological chemistry of chalcone compounds.

## Figures and Tables

**Table 1:** Group of chalcone analogues with ring A as phenothazine moiety.

No.	Structure of Phenothiazinyl Chalcones	Ring B				Ref.
		R_1_	R_2_	R_3_	R_4_	
**1**	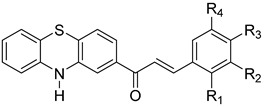	H	H	N(CH_3_)_2_	H	<N> ^1^
**2**		H	H	OCH_2_C_6_H_5_	H	<N> ^1^
**3**		H	Br	H	H	<N> ^1^
**4**		H	H	Cl	H	[31]
**5**		H	H	H	H	[32]
**6**		H	H	OCH_3_	H	[31]
**7**		Cl	H	H	H	<N> ^1^
**8**		OCH_3_	H	OCH_3_	H	<N> ^1^
**9**		2-thiophenyl				<N> ^1^

^1^ <N>: new compound.

**Table 2:** Group of chalcone analogues with ring A as thiophene/furan/pyridine/phenyl moieties.

No.	Chalcone Structure	Ring A	Ring B				Ref.
		X	R_1_	R_2_	R_3_	R_4_	
**10**	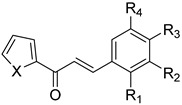	S	H	H	Cl	H	[32]
**11**		S	OH	H	H	H	[33]
**12**		S	H	H	N(CH_3_)_2_	H	[33]
**13**		S	H	OCH_3_	OCH_3_	OCH_3_	[33]
**14**		O	H	OCH_3_	OCH_3_	OCH_3_	[33]
**15**		O	H	H	N(CH_3_)_2_	H	[33]
**16**	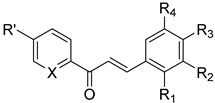	R′: H; X: N	H	OCH_3_	OCH_3_	OCH_3_	[33]
17 *		R′: H; X: C-OH	3-pyridyl				[34]
18 *		R′: H; X: C-OH	2-pyridyl				[34]
19 *		R′:OCH_3_; X: C-OH	3-pyridyl				[34]
20 *		R′:OCH_3_;X: C-OH	2-pyridyl				[34]

*: known compounds but their spectra data was not available.

**Table 3:** Cytotoxic profile of the potent synthesized heterocyclic chalcones on RMS cells.

Compounds	IC_50_ (μM)	Compounds	IC_50_ (μM)
**1**	Nd	**11**	48.77 ± 2.42
**2**	Nd	**12**	Nd
**3**	Nd	**13**	26.64 ± 1.42
**4**	Nd	**14**	29.06 ± 1.82
**5**	Nd	**15**	77.34 ± 4.69
**6**	Nd	**16**	19.57 ± 1.04
**7**	18.04 ± 1.63	**17**	92.85 ± 6.14
**8**	19.65 ± 1.63	**18**	91.12 ± 2.45
**9**	12.51 ± 1.74	**19**	26.62 ± 1.72
**10**	49.55 ± 3.20	**20**	20.77 ± 1.33
Paclitaxel was used as the reference			10.84 ± 0.47

**Table 4:** Cytotoxic properties of the potent synthesized heterocyclic chalcones on noncancerous LLC-PK1 cells.

Compounds	% Cell Viability on Noncancer LLC-PK1 cells		
	100 μM	50 μM	10 μM
**7**	94.8 ± 7.6	109.4 ± 10.9	100.2 ± 6.5
**8**	105.2 ± 5.4	103.2 ± 6.4	97.3 ± 10.2
**9**	97.1 ± 5.0	98.2 ± 7.3	98.8 ± 10.6
**16**	104.3 ± 6.5	101.9 ± 12.1	99.1 ± 3.1
Paclitaxel (reference)	20 μM	10 μM	2 μM
	75.0 ± 3.6	108.1 ± 8.2	103.4 ± 6.0
